# Prognostic significance of lateral pelvic lymph node dissection for middle-low rectal cancer patients with lateral pelvic lymph node metastasis: a propensity score matching study

**DOI:** 10.1186/s12885-022-09254-4

**Published:** 2022-02-03

**Authors:** Sicheng Zhou, Yujuan Jiang, Wei Pei, Jianwei Liang, Zhixiang Zhou

**Affiliations:** Department of Colorectal Surgery, National Cancer Center/National Clinical Research Center for Cancer/Cancer Hospital, Chinese Academy of Medical Sciences and Peking Union Medical College, 17 Panjiayuan South Lane, Chaoyang District, Beijing, 100021 China

**Keywords:** Rectal cancer, Later pelvic lymph node dissection, Later pelvic lymph node metastasis, Prognosis, Region

## Abstract

**Background:**

There is still controversy regarding the clinical value and significance of lateral pelvic lymph node (LPN) dissection (LPND). The present study aimed to investigate whether the addition of LPND to total mesorectal excision (TME) confers survival benefits in rectal cancer patients with clinical lateral pelvic node metastasis (LPNM).

**Methods:**

From January 2015 to January 2021, a total of 141 rectal cancer patients with clinical evidence of LPNM who underwent TME + LPND were retrospectively analysed and divided into the LPNM group (*n* = 29) and the non-LPNM group (n = 112). The LPNM group was further subdivided into a high-risk LPNM group (*n* = 14) and a low-risk LPNM group (*n* = 15). Propensity score matching (PSM) was performed to minimize selection bias. The primary outcomes of this study were 3-year overall survival (OS) and disease-free survival (DFS).

**Results:**

Of the 141 patients undergoing LPND, the local recurrence rate of patients with LPNM was significantly higher than that of patients without LPNM both before (27.6% vs. 4.5%, *P* = 0.001) and after (27.6% vs. 3.4%, *P* = 0.025) PSM. Multivariate analysis revealed that LPNM was an independent risk factor for not only OS (HR: 3.06; 95% CI, 1.15–8.17; *P* = 0.025) but also DFS (HR: 2.39; 95% CI, 1.18–4.87; *P* = 0.016) in patients with LPNM after TME + LPND. When the LPNM group was further subdivided, multivariate logistic regression analysis showed that OS and DFS were significantly better in the low-risk group (obturator/internal iliac artery region and < 2 positive LPNs).

**Conclusion:**

Even after LPND, LPNM patients have a poor prognosis. Moreover, LPNM is an independent poor prognostic factor affecting OS and DFS after TME + LPND. However, LPND appears to confer survival benefits to specific patients with single LPN involvement in the obturator region or internal iliac vessel region. Furthermore, LPND may have no indication in stage IV patients and should be selected carefully.

## Introduction

The lateral lymph node metastasis (LPNM) pathway of middle and low rectal cancer was first proposed by Gerota in 1895 [1], and the anatomical theoretical system of lateral pelvic lymphatic drainage of rectal cancer gradually formed in the 1950s [2]. LPNM has been reported in approximately 16–23% of patients with middle to low rectal cancer [3], and it is an important predictive factor for local recurrence and long-term survival [4, 5]. Lateral pelvic lymph node dissection (LPND), as a potential radical surgery, is still controversial worldwide. In Western countries, LPNM (except internal iliac lymph nodes) is considered a systemic disease. Even if LPND is performed, the five-year survival rate is only 20–45% [6], and it may increase the possibility of sexual function and urinary dysfunction. Therefore, NCCN guidelines and ESMO guidelines recommend neoadjuvant chemoradiotherapy (nCRT) combined with total mesorectal resection (TME) as the standard treatment mode for advanced rectal cancer, rather than prophylactic LPND alone [7]. However, in Japan, the lateral pelvic lymph nodes (LPNs) in the area of the obturator, external iliac, and common iliac were regarded as regional lymph nodes, which were considered within the scope of the N3 stage. The JSCCR guidelines clearly indicate that prophylactic LPND should be performed for patients with T3-T4 rectal cancer that is below the peritoneal reflection [8, 9]. However, the level of evidence is relatively low, and thus, this procedure is not widely implemented.

Several Japanese studies have suggested that the overall benefit related to local recurrence and survival of LPND is not promising in patients with LPNM [10–14]. Therefore, it is necessary to clarify the effectiveness of LPND with regard to increasing local control and prolonging survival. Therefore, we designed a retrospective cohort study to investigate the prognostic significance related to the local control effect and survival benefit of LPND in rectal cancer patients with clinical evidence of LPNM and to explore the types of patients with LPNM that could receive some prognostic benefit from LPND.

## Materials and methods

### Patients and methods

**Fig. 1:**
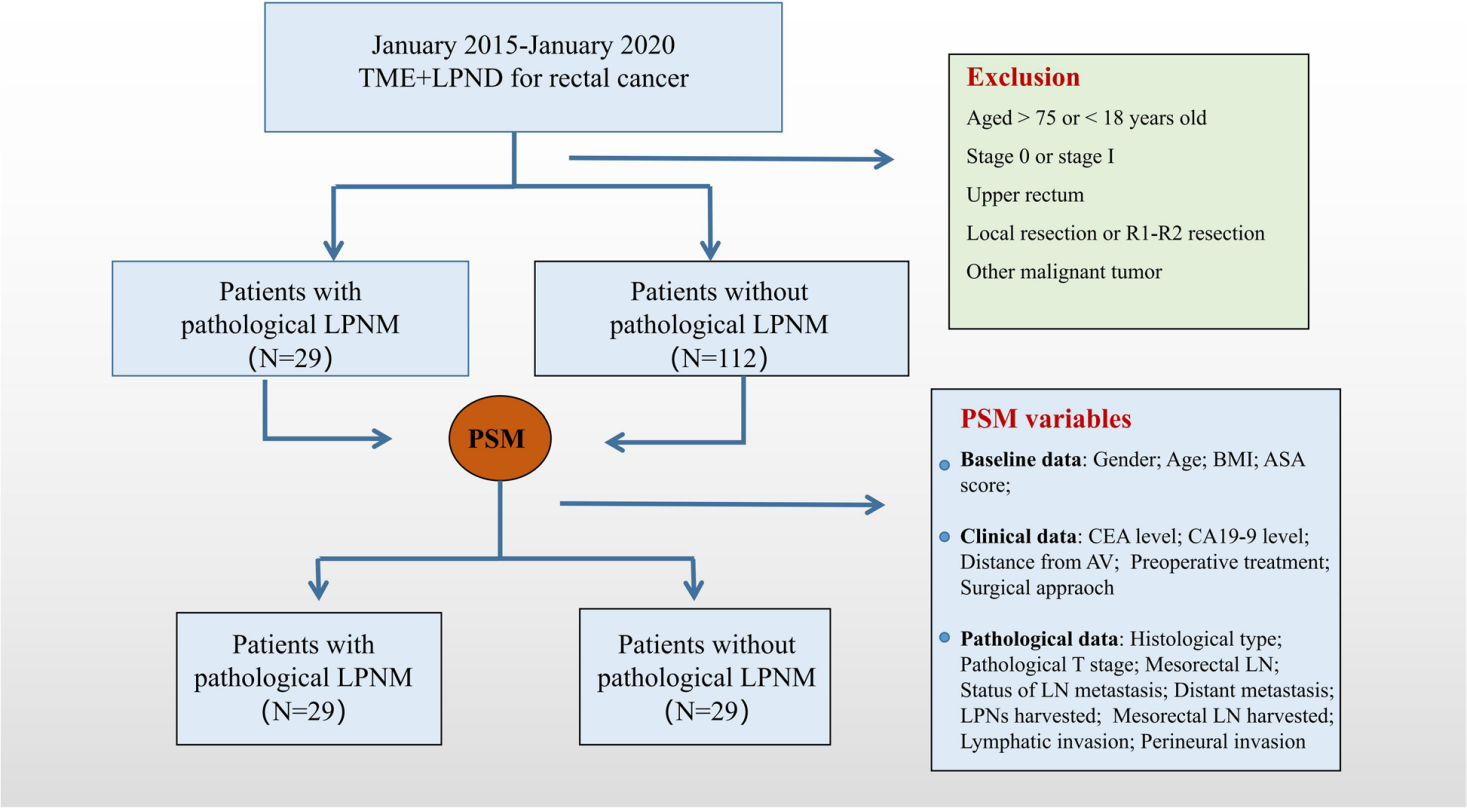
Group flow chart

Rectal cancer patients with clinical evidence of LPNM who underwent TME + LPND at the National Cancer Center/National Clinical Research Center for Cancer/Cancer Hospital, Chinese Academy of Medical Sciences and Peking Union Medical College between January 2015 and January 2021 were identified and reviewed. The inclusion criteria were as follows: (1) pathology confirmed as adenocarcinoma; (2) lower margin of the tumour located below the peritoneal reflection; and (3) clinically advanced rectal cancer (cT3-T4/cN +). The exclusion criteria were as follows: (1) patients with a history of other malignancies; and (2) patients who underwent local resection or R1-R2 resection. Finally, a total of 141 cases were included and divided into the LPNM group (*n* = 29) and the non-LPNM group (*n* = 112) according to the pathological results. All enrolled patients were included in the propensity score matching (PSM) process, and 29 matched pairs were eventually selected (Fig. 1). This retrospective study was approved by the Ethics Committee of the Cancer Hospital, Chinese Academy of Medical Sciences (NCC 2017-YZ-026, Oct 17, 2017). All of the enrolled patients provided informed consent, and this study complied with the STROBE Guidelines.

### Diagnosis and treatment

Routine preoperative investigations for all patients included laboratory examination, endoscopy, abdominal computed tomography (CT), and pelvic magnetic resonance imaging (MRI). Clinical LPNM was diagnosed by two imaging specialists who specialized in gastrointestinal cancer based on MRI before treatment. Meeting one or more of the following criteria was considered clinically LPNM, and TME + LPND was performed: (1) short diameter of LPN > 0.8 cm; (2) inhomogeneous or intense enhancement; and (3) irregular shape with rough edges. Tumour staging was performed using the American Joint Committee on Cancer (AJCC) staging system (8th edition) [15]. Treatment strategies for each patient were determined by multidisciplinary team meetings (MDTs) that incorporated radiologists and medical and surgical oncologists. Postoperative complications were categorized according to the Clavien-Dindo classification [16]. According to the guidelines of the NCCN, all patients with pT3/T4 or N + underwent adjuvant chemotherapy postoperatively.

### Subgrouping of LPNM

Twenty-nine patients in the LPNM group were further divided into two groups based on the distant metastasis, actual number and region of LPNMs. According to the JSCCR guidelines, LPNs were divided into 5 regions: the common iliac vessel region, the proximal iliac vessel region, the distal iliac vessel region, the obturator region and the external iliac vessel region [8]. Patients with < 2 positive LPNs, with positive LPNs in the obturator or internal iliac artery region and without distant metastasis were assigned to the low-risk LPNM group (*n* = 15), and patients without any of these factors were assigned to the high-risk LPNM group (*n* = 14) (Table1).

**Table 1:** Grouping criteria for LPNM

Characteristic	High-risk group (*n* = 14)	Low-risk group (*n* = 15)
LPNs Location		
Obturator or the external iliac vessel region	4 (28.6)	15 (100.0)
Other	10 (71.4)	0 (0)
Distant metastasis		
Presence	4 (28.6)	0 (0)
Absence	10 (71.4)	15 (100.0)
The number of LPNs		
< 2	5 (35.7)	15 (100.0)
≥ 2	9 (64.3)	0 (0)

### LPND procedure

All patients were treated with standard TME and LPND with laparoscopic or open procedures. Based on preoperative MRI evaluation, therapeutic unilateral or bilateral LPND was performed. Bilateral LPND is not performed routinely unless preoperative MRI suggests bilateral LPNM. As we described previously [17, 18], a five-port technique was adopted. After total mobilization of the rectum and distal rectal transection according to the TME principle, unilateral or bilateral LPND was performed appropriately. The common iliac vessel, external iliac vessel, internal iliac and obturator lymph nodes were dissected. The internal iliac vessels are routinely preserved during dissection. During LPND, the ureter, hypogastric nerves and obturator nerve were carefully identified and preserved.

### Follow-up

After the operation, all patients were followed up by telephone or outpatient visits until death due to recurrence or metastasis of rectal cancer or February 1, 2021, whichever came first. The follow-up examination consisted of serum tumour marker measurements, abdominal CT, and pelvic MRI 3–6 months for the first three years and every 6 months for the next two years. The long-term endpoints of this study were 3-year overall survival (OS) and disease-free survival (DFS), and the data were collected based on this follow-up survey.

### Statistical analysis

Clinical and pathological factors are expressed as frequencies and percentages or means ± standard deviations and were analysed separately using the χ^2^ test or Fisher’s exact test and the t test. PSM was performed by logistic regression to reduce the imbalance in these 2 groups. The matching ratio was 1:1, and the covariates included age, sex, body mass index (BMI), CEA level, CA19-9 level, American Society of Anesthesiologists (ASA) category, preoperative treatment, distant metastasis, surgical approach, histology, T stage, N stage, perineural invasion, lymphatic invasion, and vascular invasion. OS and DFS were calculated by the Kaplan–Meier method and compared by the log–rank test. The variables determined to have a *P* value < 0.05 in univariate analysis were subsequently tested by multivariate analysis through a Cox regression model, and an odds ratio with a 95% confidence interval was calculated for each variable. A *P* value < 0.05 was considered statistically significant. Statistical analysis was performed using SPSS for Windows version 20.0 (SPSS, Chicago, Illinois, USA).

## Results

### Clinical and pathological characteristics

**Fig. 2:**
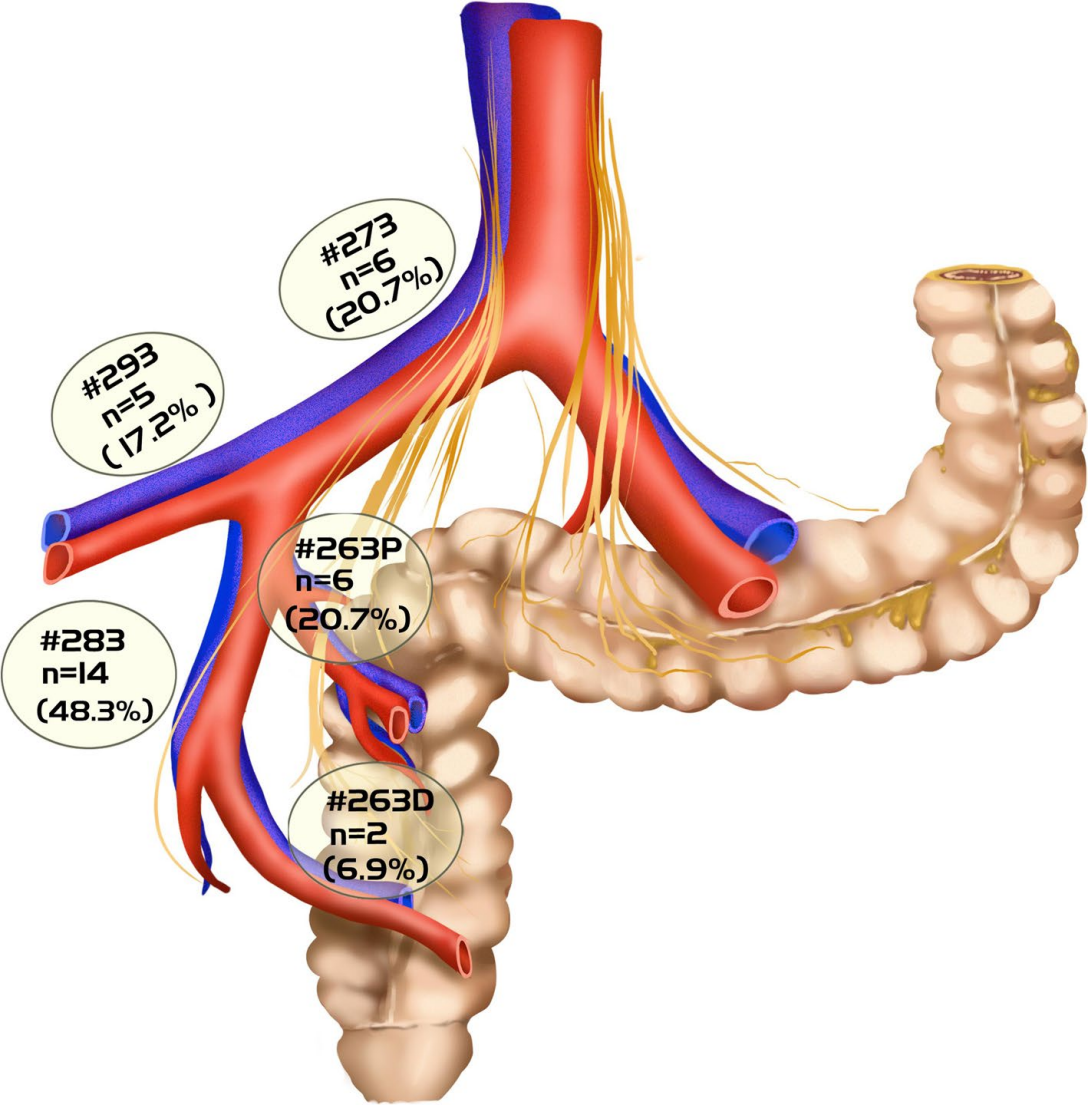
Distribution of lateral lymph node metastases of 29 patients in the LPNM group. #263P (proximal internal iliac lymph nodes); #263D (distal internal iliac lymph nodes); #273 (common iliac lymph nodes); #283 (lymph nodes around obturator); and #293 (external iliac lymph nodes)

Of 141 patients with rectal cancer and clinical LPNM, 29 (20.6%) patients were postoperatively diagnosed with pathological LPNM by pathology. The lymph nodes around the obturator were the most common LPN metastatic site (*n* = 14, 48.3%), followed by the internal iliac region (*n* = 8, 27.6%), the common iliac region (*n* = 6, 20.7%) and the external iliac region (*n* = 5, 17.2%) (Fig. 2).

The clinicopathological characteristics are listed in Table 2. After matching, the LPNM group and non-LPNM group were well balanced in terms of age, sex, BMI, CEA level, CA19-9 level, ASA category, preoperative treatment, distant metastasis, surgical approach, histology, pT stage, pN stage, perineural invasion, lymphatic invasion, and vascular invasion (*P* > 0.05).

**Table 2:** Clinical and pathological characteristics of 141 rectal cancer patients with or without pathological LPNM before and after matching

Variables	Original cohort			Matched cohort		
	LPNM (*n* = 29)	Non-LPNM (*n* = 112)	*P*	LPNM (*n* = 29)	Non-LPNM (*n* = 29)	*P*
Age (years, mean ± SD)	57.5 ± 11.7	56.2 ± 10.4	0.551	57.5 ± 11.7	58.6 ± 10.1	0.632
Gender			0.462			0.594
Male	18 (62.1)	61 (54.5)		18 (62.1)	16 (55.2)	
Female	11 (37.9)	51 (45.5)		11 (37.9)	13 (44.8)	
BMI (kg/m^2^, mean ± SD)	24.3 ± 3.0	25.0 ± 3.2	0.295	24.3 ± 3.0	24.5 ± 2.5	0.930
CEA level (ng/mL, mean ± SD)	15.0 ± 32.0	8.4 ± 15.1	0.124	15.0 ± 32.0	12.9 ± 22.5	0.811
CA19-9 level (ng/mL, mean ± SD)	47.9 ± 101.8	19.0 ± 17.3	0.169	47.9 ± 101.8	35.2 ± 41.4	0.373
ASA category			0.580			1.000
I-II	26 (89.7)	106 (94.6)		26 (89.7)	27 (93.1)	
III-IV	3 (10.3)	6 (5.4)		3 (10.3)	2 (6.9)	
Preoperative treatment	16 (55.2)	58 (51.8)	0.745	16 (55.2)	17 (58.6)	0.791
Distant metastasis	4 (13.8)	8 (7.2)	0.441	4 (13.8)	4 (13.8)	1.000
Surgical approach			0.651			1.000
Open	1 (3.4)	9 (8.0)		1 (3.4)	2 (6.9)	
Laparoscopic	28 (96.6)	103 (92.0)		28 (96.6)	27 (93.1)	
Histology			0.047			0.588
Moderate	16 (55.2)	83 (74.1)		17 (58.6)	19 (65.5)	
Poor/Mucinous/signet	13 (44.8)	29 (25.9)		12 (41.4)	10 (34.5)	
pT stage			0.012			0.666
T1-T2	2 (6.9)	33 (29.5)		2 (6.9)	4 (13.8)	
T3-T4	27 (93.1)	79 (70.5)		27 (93.1)	25 (86.2)	
pN stage			< 0.001			0.636
N0	3 (10.3)	56 (50.0)		3 (10.3)	5 (17.3)	
N1	12 (41.4)	33 (29.5)		12 (41.4)	13 (44.8)	
N2	14 (48.3)	23 (20.5)		14 (48.3)	11 (37.9)	
Perineural invasion	14 (48.3)	41 (36.6)	0.251	14 (48.3)	12 (41.4)	0.597
Lymphatic invasion	13 (44.8)	28 (25.0)	0.036	13 (44.8)	11 (37.9)	0.594
Vascular invasion	13 (44.8)	33 (29.5)	0.116	13 (44.8)	10 (34.5)	0.421
Mesorectal lymph nodes harvested	15.6 ± 8.2	18.7 ± 10.2	0.137	15.6 ± 8.2	17.3 ± 9.6	0.691
LPLNs harvested	9.3 ± 5.5	9.9 ± 6.1	0.773	9.3 ± 5.5	10.9 ± 6.4	0.573

### Operative and perioperative data

Operative and perioperative data are shown in Table 3. Patients in both groups had comparable types of operations, LPND, operative time, estimated blood loss, postoperative complications, time to first flatus, and postoperative hospital stay before and after matching (*P* > 0.05). No deaths were recorded during the perioperative period in either group.

**Table 3:** Perioperative outcomes of 141 rectal cancer patients with or without pathological LPNM before and after matching

Variables	Original cohort			Matched cohort		
	LPNM (*n* = 29)	Non-LPNM (*n* = 112)	*P*	LPNM (*n* = 29)	Non-LPNM (*n* = 29)	*P*
Types of operation (%)			0.428			0.883
Low anterior resection	11 (37.9)	54 (48.2)		11 (37.9)	10 (34.5)	
Abdominoperineal resection	16 (55.2)	55 (49.1)		16 (55.2)	16 (55.2)	
Hartmann procedure	2 (6.9)	3 (2.7)		2 (6.9)	3 (10.3)	
LPND			0.997			0.517
Unilateral dissection	22 (75.9)	85 (75.9)		22 (75.9)	24 (82.8)	
Bilateral dissection	7 (24.1)	27 (24.1)		7 (24.1)	5 (17.2)	
Operative time, min (mean ± SD)	275.4 ± 72.8	265.8 ± 76.5	0.542	275.4 ± 72.8	283.3 ± 77.2	0.680
Estimated blood loss, ml (mean ± SD)	83.1 ± 61.9	84.3 ± 108.7	0.955	83.1 ± 61.9	80.1 ± 91.4	0.872
Postoperative complications (Grade2-4)	4 (13.8)	21 (18.8)	0.533	4 (13.8)	5 (20.7)	0.487
Postoperative bleeding	0 (0)	2 (1.8)		0 (0)	0 (0)	
Ileus	1 (3.4)	2 (1.8)		1 (3.4)	1 (3.4)	
Anastomosis leakage	0 (0)	3 (2.7)		0 (0)	1 (3.4)	
Pelvic cavity abscess	1 (3.4)	2 (1.8)		1 (3.4)	1 (3.4)	
Pneumonia	1 (3.4)	8 (7.1)		1 (3.4)	3 (10.3)	
Wound infection	1 (3.4)	4 (3.6)		1 (3.4)	1 (3.4)	
Urinary retention	0 (0)	2 (1.8)		0 (0)	0 (0)	
Time to first flatus (day, mean ± SD)	3.1 ± 1.3	3.1 ± 1.4	0.868	3.1 ± 1.3	3.3 ± 1.6	0.683
Postoperative hospital stay (day, mean ± SD)	8.9 ± 4.5	8.7 ± 5.1	0.872	8.9 ± 4.5	9.3 ± 5.6	0.811
Re-operation	0 (0)	1 (0.9)	1.000	0 (0)	0 (0)	-
Mortality	0 (0)	0 (0)	-	0 (0)	0 (0)	-

### Postoperative recurrence pattern

Postoperative recurrence is shown in Table 4. The postoperative overall recurrence rate (51.7% vs. 21.4%, *P* = 0.001) and local recurrence rate (27.6% vs. 4.5%, *P* = 0.001) were significantly higher in the LPNM group than in the non-LPNM group before matching. After eliminating confounding factors through matching, patients in the LPNM group still had significantly higher local recurrence rates (27.6% vs. 3.4%, *P* = 0.025).

**Table 4:** Postoperative recurrence of 141 rectal cancer patients with or without pathological LPNM before and after matching

Variables	Original cohort			Matched cohort		
	LPNM (*n* = 29)	Non-LPNM (*n* = 112)	*P*	LPNM (*n* = 29)	Non-LPNM (*n* = 29)	*P*
Overall recurrence (%)	15 (51.7)	24 (21.4)	0.001	15 (51.7)	8 (27.6)	0.060
Local recurrence	8 (27.6)	5 (4.5)	0.001	8 (27.6)	1 (3.4)	0.025
Distant metastasis	8 (27.6)	19 (17.0)	0.195	8 (27.6)	7 (24.1)	0.764
Liver metastasis	5 (17.2)	11 (9.8)		5 (17.2)	5 (17.2)	
Lung metastasis	1 (3.4)	11 (9.8)		1 (3.4)	5 (17.2)	
Bone metastasis	2 (6.9)	2 (1.8)		2 (6.9)	2 (6.9)	
Peritoneal metastasis	1 (3.4)	0 (0)		1 (3.4)	0 (0)	
Others	0 (0)	2 (1.8)		0 (0)	0 (0)	

### Survival analysis

**Fig. 3:**
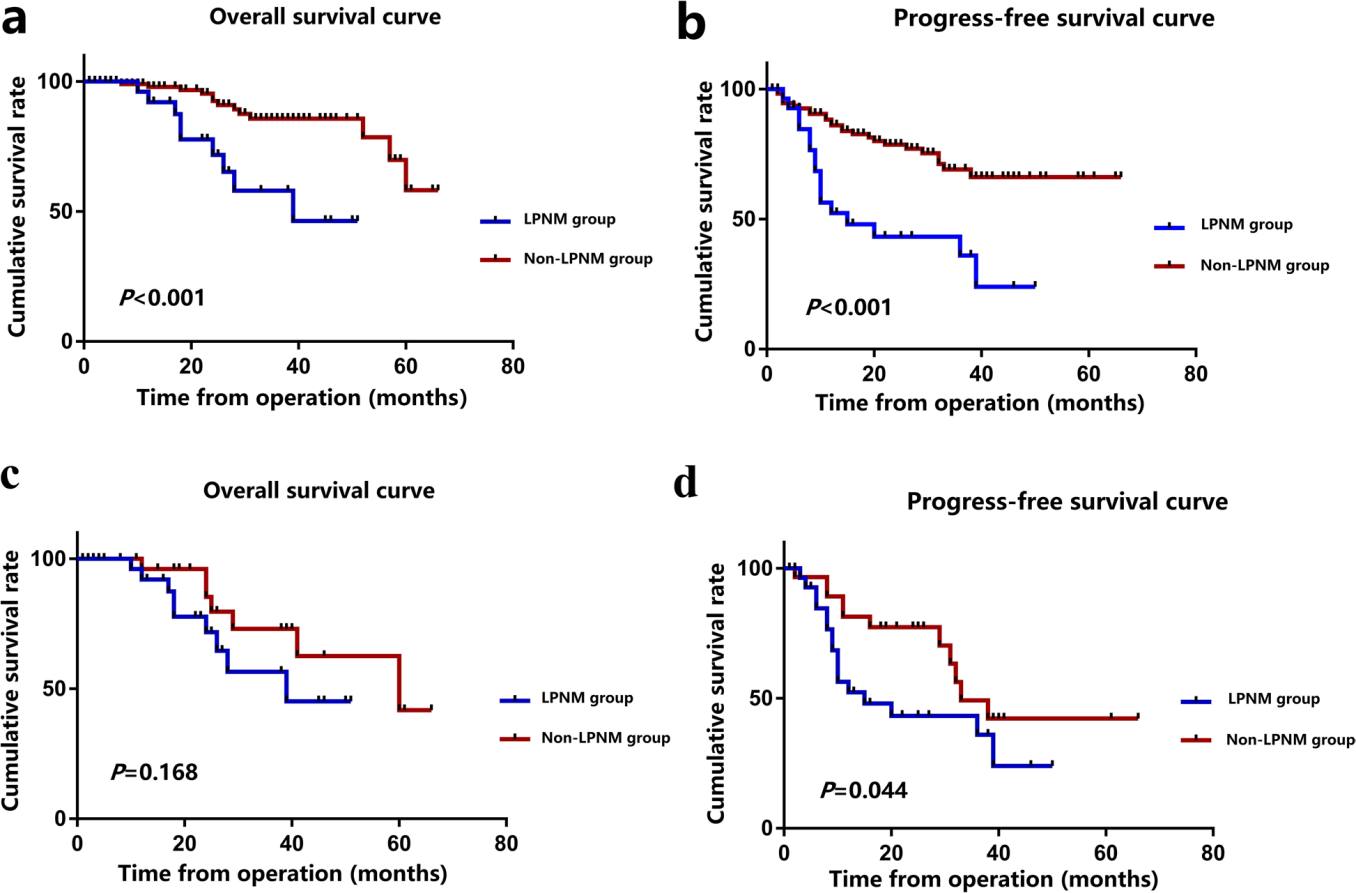
**a** Overall survival rate of patients in the LPNM group and non-LPNM group before propensity score matching. **b** Disease-free survival rate of patients in the LPNM group and non-LPNM group before propensity score matching.** c** Overall survival rate of patients in the LPNM group and non-LPNM group after propensity score matching. **d** Disease-free survival rate of patients in the LPNM group and non-LPNM group after propensity score matching

The median follow-up period of the whole group was 25.0 (range, 2–66) months. Before matching, the OS (*P* < 0.001) and DFS (*P* < 0.001) of patients in the LPNM group were significantly worse than those of patients in the non-LPNM group (Fig. 3a, b). After matching, the DFS of patients in the LPNM group was also found to be significantly worse than that in the non-LPNM group (*P* = 0.044) (Fig. 3d), while there was no significant difference in OS between the two groups (*P* = 0.168) (Fig. 3c).

**Fig. 4:**
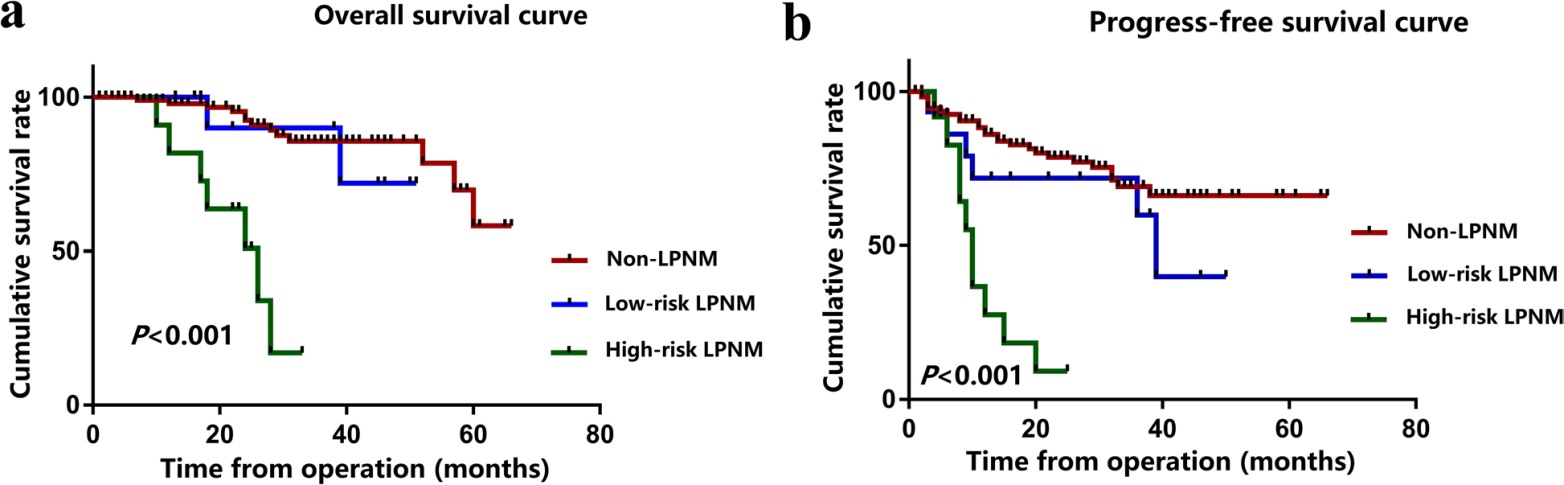
**a** Overall survival rate of patients in the high-risk LPNM group, low-risk LPNM group and non-LPNM group. **b** Disease-free survival rate of patients in the high-risk LPNM group, low-risk LPNM and non-LPNM group

Univariate and multivariate regression analyses were performed to identify prognostic factors for OS and DFS of patients with clinical LPNM who underwent TME + LPND. In univariate analysis, histology, perineural invasion, lymphatic invasion, N stage, and LPNM significantly affected OS (*P* < 0.05). In addition, DFS was significantly affected by the preoperative CEA level, perineural invasion, lymphatic invasion, N stage, and LPNM (*P* < 0.05). In multivariate regression analysis, LPNM was an independent risk factor not only for OS (HR: 3.06; 95% CI, 1.15–8.17; *P* = 0.025) but also for DFS (HR: 2.39; 95% CI, 1.18–4.87; *P* = 0.016). Moreover, lymphatic invasion was another independent risk factor for OS (HR: 3.34; 95% CI, 1.13–9.88; *P* = 0.003) (Table 5).

**Table 5:** Univariate and multivariate analyses for overall survival and disease-free survival of the 141 rectal patients with clinical LPNM who underwent TME + LPND

Variables	Overall survival				Disease-free survival			
	Univariate analysis		Multivariate analysis		Univariate analysis		Multivariate analysis	
	HR(95%CI)	P	HR(95%CI)	P	HR(95%CI)	P	HR(95%CI)	P
Gender: male/female	0.86 (0.37–1.98)	0.716			0.85 (0.46–1.56)	0.600		
Age	1.02 (0.97–1.06)	0.459			0.99 (0.97–1.03)	0.817		
Preoperative treatment	1.70 (0.65–4.48)	0.283			1.59 (0.85–3.00)	0.149		
Preoperative CEA level	1.01 (0.98–1.03)	0.587			1.01 (1.00–1.03)	0.050	1.01 (0.99–1.02)	0.165
Preoperative CA19-9 level	1.00 (0.99–1.01)	0.160			1.00 (0.99–1.01)	0.512		
Histology	2.83 (1.21–6.64)	0.017	1.38 (0.55–3.44)	0.489	1.77 (0.95–3.29)	0.073		
Perineural invasion	2.78 (1.19–6.46)	0.018	1.48 (0.54–4.09)	0.450	2.06 (1.13–3.76)	0.019	1.77 (0.86–3.65)	0.121
Vascular invasion	1.29 (0.52–3.19)	0.589			0.98 (0.50–1.91)	0.953		
Lymphatic invasion	6.00 (2.42–14.89)	< 0.001	3.34 (1.13–9.88)	0.003	2.31 (1.24–4.29)	0.008	1.48 (0.70–3.12)	0.303
T stage: T3-4/T1-2	2.36 (0.70–8.00)	0.168			1.24 (0.61–2.51)	0.557		
N stage								
N0	Reference		Reference		Reference		Reference	
N1	1.67 (0.51–5.50)	0.398	1.08 (0.30–3.89)	0.905	1.38 (0.66–2.90)	0.390	0.87 (0.38–2.03)	0.751
N2	5.06 (1.74–14.76)	0.003	2.68 (0.84–8.54)	0.096	2.32 (1.12–4.83)	0.024	1.16 (0.48–2.85)	0.741
LPN metastasis	4.49 (1.82–11.12)	0.001	3.06 (1.15–8.17)	0.025	2.95 (1.59–5.50)	0.001	2.39 (1.18–4.87)	0.016
Mesorectal LN harvested	0.98 (0.93–1.03)	0.411			0.99 (0.96–1.03)	0.698		
LPN harvested	0.99 (0.98–1.01)	0.345			1.00 (0.99–1.01)	0.264		
Adjuvant chemotherapy	0.58 (0.25–1.35)	0.209			0.72 (0.39–1.33)	0.292		
Anastomosis leakage or SSI	1.11 (0.44–3.10)	0.533			1.66 (0.23–12.06)	0.618		

Univariate and multivariate regression analyses were performed to identify prognostic factors for OS and DFS of patients with pathological LPNM. These patients were divided into a high-risk LPNM group and a low-risk LPNM group according to the site (obturator or internal iliac artery region) and number (< 2 positive LPNs) of LPNMs. The OS and DFS of the patients in the high-risk LPNM group were significantly worse than those of patients in the low-risk LPNM group and non-LPNM group (Fig. 4a, b). In univariate analysis, lymphatic invasion and high-risk LPNM significantly affected both OS and DFS (*P* < 0.05). According to multivariate analysis, high-risk LPNM was an independent risk factor affecting both OS (HR: 9.23; 95% CI, 1.46–87.35; *P* = 0.032) and DFS (HR: 4.39; 95% CI, 1.33–13.16; *P* = 0.041) (Table 6).

**Table 6:** Univariate and multivariate analyses for overall survival and disease-free survival of the 29 rectal patients with pathological LPNM

Variables	Overall survival				Disease-free survival			
	Univariate analysis		Multivariate analysis		Univariate analysis		Multivariate analysis	
	HR(95%CI)	P	HR(95%CI)	P	HR(95%CI)	P	HR(95%CI)	P
Gender: male/female	0.72 (0.19–2.71)	0.625			0.99 (0.37–2.71)	0.998		
Age	1.01 (0.95–1.06)	0.811			0.99 (0.96–1.03)	0.767		
Preoperative treatment	2.73 (0.56–13.34)	0.214			1.78 (0.65–4.94)	0.265		
Preoperative CEA level	0.99 (0.93–1.05)	0.705			1.01 (1.00–1.02)	0.181		
Preoperative CA19-9 level	1.00 (0.99–1.01)	0.858			1.00 (0.99–1.01)	0.677		
Histology	0.89 (0.22–3.64)	0.870			1.00 (0.36–2.78)	0.999		
Perineural invasion	3.89 (0.34–18.10)	0.083			2.03 (0.70–5.90)	0.195		
Vascular invasion	1.06 (0.28–3.99)	0.935			0.77 (0.28–2.14)	0.612		
Lymphatic invasion	6.64 (1.35–32.78)	0.020	2.74 (0.44–17.15)	0.280	3.03 (1.01–9.10)	0.048	1.65 (0.45–5.99)	0.447
T stage: T3-4/T1-2	1.17 (0.15–9.42)	0.884			0.50 (0.14–1.80)	0.289		
N stage								
N0	Reference				Reference			
N1	0.17 (0.15–1.85)	0.145			0.62 (0.15–2.63)	0.517		
N2	1.83(0.35–9.68)	0.477			1.38 (0.34–5.57)	0.654		
Mesorectal LN harvested	0.98 (0.91–1.06)	0.638			1.00 (0.94–1.06)	0.994		
LPN harvested	1.01 (0.95–1.08)	0.742			0.98 (0.92–1.05)	0.605		
Adjuvant chemotherapy	0.69 (0.17–2.77)	0.598			0.39 (0.14–1.08)	0.071		
Anastomosis leakage or SSI	1.72 (0.26–11.53)	0.547			1.42 (0.45–3.93)	0.342		
High risk LPNM	15.33 (1.77–133.46)	0.013	9.23 ( 1.46–87.35)	0.032	4.46 (1.38–14.46)	0.013	4.39 (1.33–13.16)	0.041

## Discussion

The prognostic value and significance of LPND is controversial because LPNM represents systemic disease in Western countries, and R0 resection for tumours cannot be achieved, while LPNM is considered a regional disease amenable to surgical cure in Japan. Our study demonstrated that rectal cancer patients with clinical evidence of LPNM developed a high local recurrence rate even with TME + LPND. Moreover, LPNM is an independent poor prognostic factor affecting OS and DFS. However, specific patients with single LPN involvement in the obturator region or the internal iliac vessel region could obtain a survival benefit from TME + LPND.

This study discovered that 20.6% of patients who underwent TME + LPND were pathologically confirmed to have LPNM. Previous studies have reported LPNM rates varying from 8.6% to 18.6% [13, 19, 20], similar to our results. In addition, the most common site of LPNM was the obturator lymph node (48.3%), followed by the internal iliac lymph node (27.6%), the common iliac lymph node (20.7%), and the external iliac lymph node (17.2%), which is also consistent with previous literature reports [21].

Several studies have demonstrated that LPNs are the most common site of postoperative recurrence [13, 14, 22]. In the present study, even after TME + LPND, the postoperative overall recurrence rate (51.7% vs. 21.4%, *P* = 0.001) and local recurrence rate (27.6% vs. 4.5%, *P* = 0.001) of patients with LPNM were significantly higher than those of patients without LPNM. After the elimination of confounding factors by PSM, the local control effect of LPND for patients with LPNM was still worse (27.6% vs. 3.4%, *P* = 0.025). A retrospective study involving 899 colorectal cancer patients at a high-volume cancer centre in Japan conducted by Wang et al. revealed that even with LPND, patients with LPNM still showed an elevated risk of local recurrence (30.0% vs. 10.0, *P* = 0.025) [13]. Similarly, Numata et al. suggested that additional LPND based on TME cannot achieve obvious local control compared with TME alone (27.8% vs. 26.4%, *P* = 1.000), while increasing the R0 resection rate is crucial to maximizing the potential merits of LPND [14]. The literature has shown that both chemotherapy and TME combined with LPND have the same long-term survival outcomes in rectal cancer patients with LPNM and that even the former can achieve a reduction in local recurrence [23]. Therefore, we suggest that LPND alone is not sufficient to achieve local control, and comprehensive treatment methods, including chemoradiotherapy during the perioperative period, should be considered to confer overall survival benefits for rectal cancer patients with LPNM.

We investigated prognostic factors in 141 patients with TME + LPND, and the results showed that the OS and DFS of patients with LPNM were significantly poorer even after LPND and that LPNM was an independent predictive value affecting OS (HR: 3.06; 95% CI, 1.15–8.17; *P* = 0.025) and DFS (HR: 2.39; 95% CI, 1.18–4.87; *P* = 0.016). Similarly, Sato et al. also proved that LPNM results in a higher recurrence rate and a poor prognosis after LPND in patients with rectal carcinoma below the peritoneal reflection [12]. The above results suggested that the potential benefits of routine use of LPND are limited or even ineffective. It has been reported in the literature that LPND may provide survival benefits for patients with certain specific LPN involvement [12, 24–26]. Yokoyama et al. classified LPNs according to the actual number and region of LPNMs and found that LPND is an effective treatment for patients with a single LPNM in the internal iliac vessel region or the obturator region [24]. Moreover, Ueno and colleagues considered that the internal iliac vessel region and the obturator region are “vulnerable fields”, and the value of LPND can be effectively assessed by estimating the nodal diameter in this “vulnerable field” [26]. Similar to the above literature, our study also demonstrated that patients in the low-risk group (obturator/internal iliac artery region and < 2 positive LPNs) achieved better survival benefits in terms of OS and DFS. The 8th edition of the AJCC indicated that lymph nodes around the internal iliac artery should be regarded as regional lymph nodes for rectal cancer, and the N stage should be included in staging considerations, which also supports our results to a certain extent.

There are several potential limitations to this study that should be considered. First, the accuracy of MRI in the diagnosis of LPNM was only 20.6% (29/141), which was related to our relatively loose diagnostic criteria for clinical LPNM. Therefore, we do not recommend that this imaging diagnostic standard be used in clinical practice, as it could result in a high false positive rate. The second potential limitation is the retrospective nature of this study, and only 141 patients were included; in particular, only 29 patients with pathological LPNM were included in the prognostic analysis, which may have caused some bias. However, we conducted PSM according to clinical and pathological characteristics to minimize selection bias. Despite these limitations, we believe that our results will improve the understanding of issues related to LPND and provide a basis for the management of LPNM in clinical practice.

## Conclusion

Our data demonstrated that even after performing LPND, patients with LPNM still have a poor long-term survival. Moreover, LPNM was found to be an independent poor prognostic factor affecting OS and DFS in patients with LPNM. However, LPND appears to confer survival benefits to specific patients with single LPN involvement in the obturator region or internal iliac vessel region. Furthermore, LPND may have no indication in stage IV patients and should be selected carefully.

## Data Availability

The data supporting the findings of this study are available on reasonable request from the corresponding author. The data are not publicly available due to privacy and ethical restrictions.

## References

[1] Gerota D (1895). Die lymphgefasse des rectums und des anus. Arch Anat Physiol.

[2] Sauer I, Bacon HE (1952). A new approach for excision of carcinoma of the lower portion of the rectum and anal canal. Surg Gynecol Obstet.

[3] Hashiguchi Y, Muro K, Saito Y (2020). Japanese Society for Cancer of the Colon and Rectum (JSCCR) guidelines 2019 for the treatment of colorectal cancer. Int J Clin Oncol.

[4] Yagi R, Shimada Y, Kameyama H (2016). Clinical significance of extramural tumor deposits in the lateral pelvic lymph node area in low rectal cancer: a retrospective study at two institutions. Ann Surg Oncol.

[5] Homma Y, Hamano T, Otsuki Y (2015). Total number of lymph node metastases is a more significant risk factor for poor prognosis than positive lateral lymph node metastasis. Surg Today.

[6] Van Gijn W, Marijnen CA, Nagtegaal ID (2011). Pre-operative radiotherapy combined with total mesorectal excision for resectable rectal cancer: 12-year follow-up of the multicentre, randomised controlled TME trial. Lancet Oncol.

[7] Bosset JF, Calais G, Mineur L (2014). Fluorouracil-based adjuvant chemotherapy after pre-operative chemoradiotherapy in rectal cancer: long-term results of the EORTC 22921 randomised study. Lancet Oncol.

[8] Yagi R, Shimada Y, Kameyama H (2016). Clinical significance of extramural tumor deposits in the lateral pelvic lymph node area in low rectal cancer: a retrospective study at two institutions. Ann Surg Oncol.

[9] Fujita S, Mizusawa J, Kanemitsu Y (2017). Mesorectal Excision With or Without Lateral Lymph Node Dissection for Clinical Stage II/III Lower Rectal Cancer (JCOG0212): a multicenter, randomized controlled. Noninferiority Trial Ann Surg.

[10] Kobayashi H, Mochizuki H, Kato T (2009). Outcomes of surgery alone for lower rectal cancer with and without pelvic sidewall dissection. Dis Colon Rectum.

[11] Akiyoshi T, Watanabe T, Miyata S (2012). Japanese society for cancer of the colon and rectum. Results of a Japanese nationwide multi-institutional study on lateral pelvic lymph node metastasis in low rectal cancer: is it regional or distant disease?. Ann Surg.

[12] Sato H, Maeda K, Maruta M (2011). Prognostic significance of lateral lymph node dissection in node positive low rectal carcinoma. Int J Colorectal Dis.

[13] Wang L, Hirano Y, Heng G (2021). The significance of lateral lymph node metastasis in low rectal cancer: a propensity score matching study. J Gastrointest Surg..

[14] Numata M, Tamagawa H, Kazama K (2021). Lateral lymph node dissection for mid-to-low rectal cancer: is it safe and effective in a practice-based cohort?. BMC Surg.

[15] Amin MB, Edge S, Greene F (2017). AJCC cancer staging manual.

[16] Clavien PA, Barkun J, de Oliveira ML (2009). The Clavien-Dindo classification of surgical complications: five-year experience. Ann Surg.

[17] Wang P, Zhou S, Zhou H, Liang J, Zhou Z (2019). Evaluating predictive factors for determining the presence of lateral pelvic node metastasis in rectal cancer patients following neoadjuvant chemoradiotherapy. Colorectal Dis.

[18] Zhou S, Jiang Y, Pei W (2021). Neoadjuvant chemoradiotherapy followed by lateral pelvic lymph node dissection for rectal cancer patients: A retrospective study of its safety and indications. J Surg Oncol.

[19] Ishihara S, Kawai K, Tanaka T (2017). Oncological Outcomes of Lateral Pelvic Lymph Node Metastasis in Rectal Cancer Treated With Preoperative Chemoradiotherapy. Dis Colon Rectum.

[20] Nagasaki T, Akiyoshi T, Fujimoto Y (2017). Preoperative Chemoradiotherapy Might Improve the Prognosis of Patients with Locally Advanced Low Rectal Cancer and Lateral Pelvic Lymph Node Metastases. World J Surg.

[21] Yamaoka Y, Kinugasa Y, Shiomi A (2017). Preoperative chemoradiotherapy changes the size criterion for predicting lateral lymph node metastasis in lower rectal cancer. Int J Colorectal Dis.

[22] Kinugasa T, Akagi Y, Shirouzu K (2014). Benefit of lateral lymph node dissection for rectal cancer: Long-term analysis of 944 cases undergoing surgery at a single center (1975–2004). Anticancer Res.

[23] Kim JC, Takahashi K, Yu CS (2007). Comparative outcome between chemoradiotherapy and lateral pelvic lymph node dissection following total mesorectal excision in rectal cancer. Ann Surg.

[24] Yokoyama S, Takifuji K, Hotta T (2014). Survival benefit of lateral lymph node dissection according to the region of involvement and the number of lateral lymph nodes involved. Surg Today.

[25] Akiyoshi T, Watanabe T, Miyata S (2012). Japanese Society for Cancer of the Colon and Rectum. Results of a Japanese nationwide multi-institutional study on lateral pelvic lymph node metastasis in low rectal cancer: is it regional or distant disease?. Ann Surg.

[26] Ueno H, Mochizuki H, Hashiguchi Y (2007). Potential prognostic benefit of lateral pelvic node dissection for rectal cancer located below the peritoneal reflection. Ann Surg.

